# Measuring resilience for Chinese-speaking populations: a systematic review of Chinese resilience scales

**DOI:** 10.3389/fpsyg.2024.1293857

**Published:** 2024-03-28

**Authors:** Zhenyu Tian, Kai Kuang, Steven R. Wilson, Patrice M. Buzzanell, Jinyi Ye, Xinyue Mao, Hai Wei

**Affiliations:** ^1^Department of Communication Studies, College of Wooster, Wooster, OH, United States; ^2^School of Journalism and Communication, Tsinghua University, Beijing, China; ^3^Department of Communication, University of South Florida, Tampa, FL, United States

**Keywords:** resilience, Chinese cultural contexts, cross-cultural scale adaptation, measurement, scale development, systematic review

## Abstract

**Introduction:**

Despite the rapid growth of interdisciplinary resilience research in Chinese contexts, no study has systematically reviewed individual-level measurement scales for Chinese-speaking populations. We report a systematic review of scales developed for or translated/adapted to Chinese-speaking contexts, where we assessed how widely used scales fare in terms of their psychometric qualities.

**Methods:**

Studies included in this review must have been published in peer-reviewed English or Chinese journals between 2015-2020 and included self-reported resilience scales in Chinese-speaking populations. Searches were conducted in PsycINFO, CNKI (completed in May 2021), and PubMed (completed in January 2024). We developed coding schemes for extracting relevant data and adapted and applied an existing evaluation framework to assess the most frequently used resilience scales by seven methodological criteria.

**Results:**

Analyses of 963 qualified studies suggested that Chinese resilience scales were used in a diverse range of study contexts. Among 85 unique kinds of resilience measures, we highlighted and evaluated the three most frequently used translated scales and three locally developed scales (nine scales in total including variations such as short forms). In short, resilience studies in Chinese contexts relied heavily on the translated 25-item Connor-Davidson Resilience Scale, which scored moderately on the overall quality. The locally developed Resilience Scale for Chinese Adolescents and Essential Resilience Scale received the best ratings but could use further development.

**Discussion:**

We discussed how future work may advance widely used scales, and specified seven methodological recommendations for future resilience scale development with existing and new scales in and beyond the Chinese study contexts. We further addressed issues and challenges in measuring resilience as a process and called on researchers to further develop/evaluate process measures for Chinese-speaking populations.

## 1 Introduction

Resilience has become a catch-all term for how individuals, communities, and nations cope with and adapt to disruptions, adversities, or stressors. Pioneered in developmental psychology, resilience scholarship has flourished across multiple areas of psychology and related disciplines (e.g., anthropology, communication, education, and medicine; Southwick et al., 2014; Houston and Buzzanell, 2020). Researchers have conceptualized resilience as a trait, process, and/or “positive” outcome (Southwick et al., 2014) and have offered numerous operational definitions and measures (Windle et al., 2011). Additionally, resilience scholarship has grown beyond its Western (and English-speaking) academic origins and cultural boundaries (Southwick et al., 2014), responding to critiques of earlier research being acultural/acontextual (Ungar, 2015).

Contributing to this movement are resilience studies in Chinese (speaking) contexts, such as how Chinese internet marketers’ “psychological resilience” could promote their sense of career success when shifting work conditions were complicated by the pandemic (Wang and Gao, 2022), and how, through resilience, social support buffered Chinese college students against anxiety due to experiencing prolonged lockdowns (He et al., 2022). Overall, studies in Chinese-speaking regions and cultures often attend to disruptions salient in, if not unique to, Chinese cultural contexts (e.g., left-behind children of migrant workers; academic stress associated with college entrance examination). Scholars have developed, translated, and adapted resilience measures for Chinese-speaking populations (Liu et al., 2017). However, assessing resilience in Chinese (and other non-English-speaking) contexts raises issues of translations and adaptations of the construct’s conceptualization and operationalization across cultural groups (Farh et al., 2006), compounded by the multiplicity of available measures.

Measurement reliability and validity are critical for obtaining scientifically useful information about resilience (Windle et al., 2011), and issues of contextualization must be considered when developing resilience measures across cultures (Farh et al., 2006; Southwick et al., 2014). Given the rise of interdisciplinary resilience studies in Chinese contexts since the mid-2010s and that Chinese remains the language with the second largest native-speaking population (Ethnologue, 2023),^1^ it is important to (a) identify frequently used scales in various contexts, (b) assess how widely used scales (both adapted from English and locally developed) fare in terms of their psychometric qualities, and (c) outline directions for future research on resilience measurement in Chinese contexts. In this study, we address these goals with a focus on individual-level resilience in Chinese-speaking populations and analyze peer-reviewed articles published *in either English or Chinese* from three major databases (Jackson and Kuriyama, 2019). Specifically, we focus on articles published over the six years of 2015–2020, from when a surge of resilience scholarship in Chinese contexts began to occur to the start of the COVID pandemic. We evaluate commonly used measures in Chinese contexts using a framework adapted from Windle et al. (2011), which builds on a widely cited methodological publication (Terwee et al., 2007) to organize a range of criteria for evaluating a measure’s psychometric quality. In applying their framework to cross-cultural/linguistic work, we identify additional qualities crucial for translational work. In what follows, we review conceptions of resilience and the emergence of resilience research in China, explain how we adapt Windle et al.’s (2011) framework for assessing resilience scales, and articulate our aims.

## 2 Literature review

### 2.1 Conceptualizations of resilience

Resilience research is characterized by “definitional diversity,” which has raised confusion about what resilience is and how to best characterize processes and/or outcomes of resilience (Luthar et al., 2000). Reviews written in both English and Chinese commonly discern three foci—trait, outcome, and process—for understanding resilience (e.g., Fletcher and Sarkar, 2013). The trait view treats resilience as an individual characteristic, a stable trait/set of traits, or innate qualities people possess (Block and Block, 1980; Connor and Davidson, 2003; Campbell-Sills and Stein, 2007). When people are faced with risks and adversities, specific resilience traits (e.g., ability to cope with change, persistence) function as a protective factor to enable individual adaptation and thriving. Scholars have problematized seeing resilience as a static trait for its acontextual assessments applied to complex social and cultural contexts (e.g., people facing poverty) and for the implied assumption that only some people have resilience or are resilient (Walsh, 2002; Ungar, 2004).

The outcome view conceptualizes resilience as the presence of positive developmental and/or adaptive outcomes, such as “healthy” attributes and behaviors (Werner et al., 1971), among different demographic groups that have experienced conditions commonly considered “unhealthy,” “risky,” or “adverse” (Masten and Barnes, 2018). Early work in developmental psychology reflects this approach, such as studies on the stress resistance and thriving of children living with Schizophrenic parents (Garmezy, 1974). These studies typically consider protective factors—both internal, such as personal resilient qualities (e.g., self-esteem), and external, such as environmental considerations (e.g., family support and community climate)—that enable positive adaptive responses to adversities (Richardson, 2002). In short, resilience is the presence of positive results despite difficulties, according to the outcome view.

The third view conceptualizes resilience as a “dynamic process” (Luthar et al., 2000), neither just the protective factors nor the adaptive outcome, but rather elements involved in experiencing, adapting to, and transforming adversities over time and across situations (Buzzanell, 2010; Windle, 2010; Fletcher and Sarkar, 2013). Richardson (2002) theorized that resilience begins with disruptions to “biopsychospiritual” homeostasis. An expansive term, “process” may refer to (a) underlying mechanisms by which protective factors interact with risk factors to enable adaptation (Rutter, 1990), (b) how humans as open systems successfully adapt to disturbances (Masten, 2015), and (c) ways in which individuals harness resources from contexts to sustain well-being despite difficulties (Panter-Brick and Leckman, 2013). Windle (2010) defined resilience as the process of “effectively negotiating, adapting to, or managing significant sources of stress or trauma” facilitated by “assets and resources within the individual, their life and environment” (p. 163), the nature of which may vary across the life course.

Scholars have not only identified distinct conceptualizations of resilience but also suggested guidelines for identifying appropriate resilience scales. This latter area of scholarship is less developed, as Windle et al. (2011) contended in their attempt to create a “robust evaluation framework” for (English) resilience scales (p. 2).

### 2.2 Resilience research in Chinese contexts

Resilience research in China and within geopolitical borders politically, historically, and culturally affiliated with China started in the 2000s (Yu and Zhang, 2005). Although resilience has been considered a construct developed by “Western” academics, particularly psychologists in the United States, scholars soon found similar ideas rooted in Chinese cultural and linguistic traditions. Such commonalities prompted researchers, such as Ungar (2009, 2015), to argue that although resilience research centers around Western psychological discourse, resilience phenomena manifest in universal *and* specific ways within and across cultural borders and through diverse ways of living and being.

In Chinese, resilience has been translated into “*fu yuan li*” (ability to recover), “*kang ni li*” (ability to resist adversity), “*xin li tan xing*” (emphasizing a “psychological” trait, the idea of “bouncing back,” and a sense of elasticity), and “*ren xing*” (a bendable, stretchable feature) by scholars in Mainland China, Hong Kong, and Taiwan (Liu et al., 2015). Some scholars draw from Chinese idioms, Taoist and Confucianist values, and traditional dialectical (co-dependent) views on adversity (*ni jing*) and growth or fortune to suggest that “*ren xing*”—a naturally developed term—best captures the meanings of resilience in everyday Chinese (e.g., Yu and Zhang, 2007; Hu and Gan, 2008). Diverging translations of resilience reflect conceptual inconsistencies. For example, translating resilience as “*fu yuan li*” implies an “ability” or “capacity,” whereas “*ren xing*” implies that resilience is a “trait” or “feature.”

Scholars also have translated and/or created resilience scales for use in Chinese cultural contexts. Popular scales such as the Connor–Davidson Resilience Scale (CD-RISC; Connor and Davidson, 2003) and the Resilience Scale (RS; Wagnild and Young, 1993) have been translated, validated, and sometimes adapted to Chinese contexts. These scales also have inspired the development of localized scales (e.g., Hu and Gan, 2008). Prior research, however, has not systematically assessed which Chinese resilience scales are used most commonly nor evaluated their psychometric properties.

### 2.3 Existing systematic reviews and current goals

Windle et al. (2011) provided a “robust methodological review” of English resilience measures for researchers and clinicians whose selection of instruments previously might have been “arbitrary and inappropriate” (p. 2), by applying the psychometric properties proposed by Terwee et al. (2007) to assess English resilience scales (see Table 1).^2^ Specifically, Windle et al. (2011) identified 19 measures, including 15 original measures and four variations (e.g., CD-RISC-25 vs. CD-RISC-10), and they reported ratings of these measures on their content validity, internal consistency, construct validity, reproducibility/reliability, and interpretability. These criteria offer a useful framework for the current study given its and Terwee et al.’s (2007) influence and how they further inform at least two recent systematic reviews on resilience measures (Zhou et al., 2020; Windle et al., 2022).^3^

**Table 1:** Scale evaluation rubric (adapted from Windle et al., 2011 and Terwee et al., 2007).

Property	Definition	Quality criteria
1. Content validity	The extent to which the domain of interest is comprehensively sampled by the items in the questionnaire (i.e., the extent to which the measure represents all facets of the construct under question)	“+” = 2: A clear description of measurement aim, target population, concept(s) that are being measured, and the item selection AND target population and (investigators OR experts) were involved in item selection
		“?” = 1: A clear description of the above-mentioned aspects is lacking OR only target population involved OR doubtful design or method
		“−” = 0: No target population involvement *Note:* This considers if at all the population is involved in any procedure of a study, not just item selection OR no information found on target population involvement
2. Internal consistency	The extent to which items in a (sub)scale are intercorrelated, thus measuring the same construct	“+” = 2: Factor analyses performed on adequate sample size (7* #items and > = 100) AND Cronbach’s alpha(s) calculated per dimension AND Cronbach’s alpha(s) between 0.70 and 0.95
		“?” = 1: No factor analysis OR doubtful design or method OR Cronbach’s alpha(s) for no more than half of the dimensions <0.70
		“−” = 0: Cronbach’s alpha(s) calculated for more than half of the dimensions <0.70 or > 0.95, despite adequate design and method OR no information found on internal consistency
3. Factor Structure*	For a locally developed scale, the extent to which responses fit the proposed factor structure. For a translated scale, the extent to which responses from the local population fit the original source language factor structure (i.e., conceptual subdimensions) and/or a revised fact structure adapted for the local context. *Note:* To receive the full score, the proposed factor structure of a measure must have been replicated in two or more independent samples, at least one of which uses CFA to assess measurement fit. Authors may initially perform exploratory factor analysis (EFA) to examine a measure’s factor structure in a data-driven fashion. To obtain the highest score, authors must also test the fit of a proposed model emerging from an initial EFA with a new sample using confirmative factor analysis (CFA).	“+” = 2: Proposed (*including revision*) factor structure is tested in at least two samples, at least one of which uses CFA.
		“?” = 1: For a proposed (*including revision*) factor structure, only EFA is conducted OR only a single sample is used; no replications of factor structure are reported in this or additional studies.
		“−” = 0: Insufficient information about CFA OR EFA on the proposed factor structure.
4. Construct validity	The extent to which scores on a particular questionnaire relate to other measures in a manner that is consistent with theoretically derived hypotheses concerning the concepts that are being measured.	“+” = 2: Specific hypotheses were formulated OR, similarly, the relationships between a concept of interest and other related constructs were clarified before testing AND at least 75% of the results are in accordance with these hypotheses
		“?” = 1: Doubtful design or method (e.g., no hypotheses; unspecified hypotheses; unclear descriptions of relationships)
		“−” = 0: Less than 75% of hypotheses were confirmed, despite adequate design and methods OR no information found on construct validity
5. Reproducibility-Reliability (Test–Retest)	The extent to which scores on a particular questionnaire remain stable over periods of time when they should be stable (based on the concept being assessed). Reproducibility involves both whether the rank ordering of individuals on a concept of interest remains stable over time, and whether people’s absolute level of the concept remains stable over time. For this reason, measures such as the Intraclass correlation (ICC) or weighted Kappa are more informative than a Pearson correlation (see Terwee et al., 2007).	“+” = 2: Intraclass correlation coefficient (ICC) or weighted Kappa > = 0.70 in a sample size > = 50 individuals; time interval is described and justified
		“?” = 1: Doubtful design or method (e.g., time interval not mentioned; sample size <50; reported only Pearson correlation coefficient)
		“−” = 0: ICC or weighted Kappa <0.70, despite adequate design and method OR no information found on reliability
6. Interpretability	The degree to which one can assign qualitative meaning to quantitative scores, based on such information: means and SD scores of qualitatively meaningful population groups (e.g., gender, age, health status). Additionally, a defined minimal important change (MIC) is needed for interpreting within-person change perceived to be important by patients of some intervention (Terwee et al., 2021). *Note*: MIC has not yet been applied to broad resilience research (Windle et al., 2011)	“+” = 2: Mean and SD scores presented of at least four relevant subgroups of participants and [not yet applicable to the current resilience research] MIC defined
		“?” = 1: Doubtful design or method OR less than four subgroups OR no MIC defined
		“−” = 0: No information found on interpretation
7. Cultural-Linguistic Specificity*	Whether a scale is translated from a different source-language scale or developed in a target language AND whether a scale is universal (etic) or specific to cultural context (emic) (Farh et al., 2006). *Note:* When studying a phenomenon recognized across cultural contexts, regardless of scale translation or development, the researchers should address (a) the universality and/or context-specificity of the construct and its operationalization and (b) engage with perspectives rooted in an appropriate context (universal and/or indigenous) by consulting its members and/or experts of the context, including literature.	“+” = 2: The position (i.e., the universality and specificity of a construct) for the specific approach of scale development /translation is clarified and justified AND cultural and linguistic appropriateness are addressed; thus, scale modification is performed before testing when appropriate OR the scale development and item selection processes involved experts and/or members socialized in the context(s) of interest.
		“?” = 1: Simple translation and back translation OR the researchers created a scale without seeking insights from experts and/or members socialized in the context(s) of interest.
		“−” = 0: No explanation about the translation OR item generation process.

For scoring, “+” = 2; “?” = 1; “−” = 0 (range = 0–14). “*” marks newly added properties.

Although no systematic review of individual-level resilience measures in Chinese contexts exists, a few narrative literature reviews written in Chinese provide insights for identifying and selecting measures. Specifically, Liu et al. (2017) discussed the progress, current understandings and models of resilience, and future directions of “domestic and foreign/international” resilience studies. They provided a list of commonly used scales developed in Chinese or other languages (mostly English). They also reported Cronbach’s α reliability for several listed scales. Similarly, Liu et al. (2015) reviewed popular scales organized around investigated populations (e.g., scales for children and teenagers). For each scale, the authors summarized its Cronbach’s α value, dimension(s), item numbers, whether it has been translated and validated, and study populations (e.g., students and nurses). However, as narrative reviews, these authors’ identification, selection, and evaluation of scales were not driven by a comprehensive review process.

In this project, we build on Windle et al.’s (2011) review to systematically synthesize Chinese resilience studies, extending it in two ways. First, applying it to a more recent period may reveal new trends, issues, and findings (or lingering problems) concerning resilience. Therefore, we review Chinese resilience studies published between 2015 and 2020. This time frame was informed by the search results of the first database that showed a noticeable increase in resilience research in 2016; hence, to map trends in the growing literature but in a still manageable scope, we limited the review to studies published between 2015 and 2020. Second, Windle et al. (2011) provided guidelines for resilience scales based on studies reported in English. In comparison, we address similar goals in the context of translating and developing scholarship from one language to another. We, hence, add two criteria to assess the extent to which (a) cultural and linguistic appropriateness is addressed when developing Chinese resilience scales (Farh et al., 2006) and (b) the factor structure of resilience measures in Chinese contexts is examined and replicated (see Table 1).

Our first goal is to identify the contexts (e.g., left-behind children, urban–rural migration) where resilience testing is relevant, as illuminated by included studies, as resilience is contextualized in disruptive events, and experts from multiple disciplines have emphasized how context matters for studying resilience (Southwick et al., 2014). We further aim to (a) identify the most frequently used resilience measurement scales for Chinese-speaking populations in studies from 2015 to 2020 and examine these scales’ popularity in relation to specific study contexts, and (b) address how such scales fare in terms of their psychometric properties. In so doing, we not only capture research developments that laid the foundation for the current boom in Chinese resilience scholarship but also make informed recommendations/guidelines for advancing and selecting research instruments in future.

## 3 Methods

The review process and report were guided by the Preferred Reporting Items for Systematic Review and Meta-Analysis Protocols (PRISMA; Moher et al., 2015; Page et al., 2021).

### 3.1 Eligibility criteria

Studies included in this systematic review met the following inclusion criteria. First, included articles must have been (1) published in peer-reviewed outlets between 2015 and 2020, (2) based on primary study results from the use of self-report resilience measurement scales, and (3) full-text accessible. Second, the primary language used by the population of interest in the included studies must have been Chinese.^4^ Third, included studies must have operationalized resilience as (1) a single concept, (2) multiple related subscales/sub-dimensions, or (3) part of a broader construct (e.g., positive psychological capital; Luthans et al., 2007) and was treated as an independent subscale in analyses (see Table 2).

**Table 2:** Eligibility criteria.

Criteria	Specifics, special cases, and inferences	
	Inclusion	Exclusion
**General Study Criteria** Primary research studies on human resilience at *the individual level*, published in *peer review outlets*, including *original data* at least in part based on *human participants’ self-report*. Not meta-analysis/systematic review. Not narrative literature review. Studies published between 2015 and 2020. The full report of the study must be accessible to at least one of the authors.	The reported resilience studies were primarily based on brain mapping/imaging; however, participants/patients also completed a self-report resilience scale.	**Outlets**: Theses and dissertations. Conference proceedings. Book reviews. **Studies**: Animal samples (e.g., rats). Only third-party report/rating (e.g., researcher; observation; parents) or imaging analyses (e.g., brain-mapping). **Resilience concept**: Family resilience. People’s perception of a geographic location’s resilience. Resilience of AI systems.
**Population, language, and location** Chinese speaking sample/instruments in written Chinese. Reports of studies written in English or Chinese. Note: Informed by the screening of the first database (PsycINFO), we focus only on studies based on *Mainland Chinese, Hong Kong, Macao,* and/or *Taiwanese* samples. These are the categories used in studies, which may intertwine for historical and political reasons but are not the concern of this systematic review.	The author(s) did not clarify, but the study very likely has used resilience measurement scales in (traditional or simplified) Chinese, or the population of interest very likely is Chinese speaking based on the given information, such as when the participants were natives of Shandong or Hong Kong.	The author(s) did not clarify, but the research population was very unlikely to speak Chinese based on the given information, such as studies from regions that do not have the tradition of using Chinese (e.g., Greece; South Africa) as its primary language. Not enough information for determining the study population and/or language because neither detail was reported. Chinese study but written neither in English nor Chinese.
**Scale specifics** Employment of *self-report* resilience measurement scale(s) that *can be identified* by the given information. *Note*: This includes popular resilience scales developed in foreign and/or native contexts (e.g., CD-RISC; RS; RSA; RSCA), less commonly used resilience scales, as well as resilience measures created specifically for the given study/studies. “Resilience measure” is operationalized as a *multiple-item* scale where: (a) The whole scale was conceptualized as measuring resilience as one concept or by multiple related concepts/subscales/sub-dimensions; (b) Resilience is a part/dimension of the larger measure (e.g., PsyCap) but either is the only subscale used or is analyzed independently.	Used seven questions derived from the Positive and Negative Affect Schedule to measure resilience. Used three- or six-item resilience sub-scale from the PsyCap Questionnaire. Used the Positive Psychological Capital Questionnaire as a whole, but the resilience score is independently discussed in relation to other variates (hope; self-efficacy; left-behind experience). Took “a multivariate approach” to measure resilience by combining academic performance, mental health and prosocial behavior. Resilience measured by another conceptually related scale (e.g., resilience measured by the Children’s Hope Scale) Concept(s) used interchangeably with resilience *in the given study* (e.g., dispositional hardiness).	Measured resilience with one single item. Isolated sub-scale/dimension of a widely used resilience scale (e.g., personal competence from RS) and no longer analyzed resilience as a whole. Used PsyCap questionnaire, which has a resilience sub-scale but only the overall scoring of PsyCap was discussed. Studied and analyzed factors (protective; risk) conceptually related to resilience (e.g., home-learning environment) in relation to other phenomena (e.g., Concerns about children’s behaviors) but did not directly measure resilience itself. In Chinese, generically referred to the instrument as “resilience scale” without providing enough information for identifying the specific instrument that was used (e.g., no citation).

### 3.2 Databases search

A systematic search was first conducted between July 2020 and May 2021 in two databases. For studies published in English, we chose PsycINFO for its coverage of more than 2,000 journals across multiple related disciplines (e.g., psychology, health, sociology, management, and communication). We then used China National Knowledge Infrastructure (CNKI) to access reports published in Chinese. CNKI is the largest academic database in China containing a wide range of sources from all disciplines, with its built-in search system enabling more targeted searches (e.g., considering quality and topics). To ensure our systematic review was comprehensive, we conducted an additional search in PubMed in January 2024. In the early scoping stage, the first author emailed six researchers cited in other reviews or studies for obtaining original scales in Chinese not available through databases, two of whom responded.

In PsycINFO and PubMed, the Boolean search query “(resilience OR resiliency OR resilient) AND (China OR Chinese)” was used. Filters applied to PsycINFO included “Linked Full Text” and “Peer Reviewed.” The first PsycINFO search was in July 2020 and was completed by another search in May 2021, with an added date limiter “2020/07/01–2020/12/31” for completing 2020 results. Based on learned experience, additional filers were added for PubMed results: “Humans,” “Chinese,” and “English,” and published between “2015/01/01” and “2020/12/31.” The search query in CNKI, with the adjusted time frame, was informed by existing conceptual reviews (e.g., Yu and Zhang, 2005) that identified commonly used translations of “resilience” (i.e., “‘心理弹性’ + ‘心理韧性’ + ‘复原力’ + ‘抗逆力’”). Specifically, “弹性” and “韧性” were both common words (e.g., in mechanical engineering and biology). We included the modifier “心理 (psychology/ical)” to avoid retrieving numerous irrelevant records (e.g., resilience of mechanic systems). We then limited the search by discipline. For example, we unselected “basic science” (e.g., physics), “engineering,” and “agriculture” while only keeping relevant ones such as healthcare, humanities, social sciences, communication, and management. To ensure quality and consistency with the peer-reviewed work in (primarily) English databases, we further filtered the search using CNKI’s built-in citation index-based qualifying system (SCI, EI, PKU Core, CSSCI, and CSCD) for academic journal publications.

### 3.3 Selection

We began by screening PsycINFO records/abstracts, which were coded for whether it (a) named a specific resilience scale (e.g., CD-RISC), (b) explicitly described measuring resilience, (c) clearly used Chinese-speaking sample(s), or (d) was clearly unqualified (e.g., mouse models, systematic reviews, and qualitative reports). To be prudent, full-text articles were assessed if there was any indication that a study used a resilience measure. These abstracts usually mentioned at least one of the following: (a) a specific resilience scale, (b) some relationship between resilience and other variables, (c) studies on phenomena often conceptually related to resilience (e.g., post-traumatic growth) despite missing the word “resilience,” and (d) resilience as a keyword.

**Figure 1:**
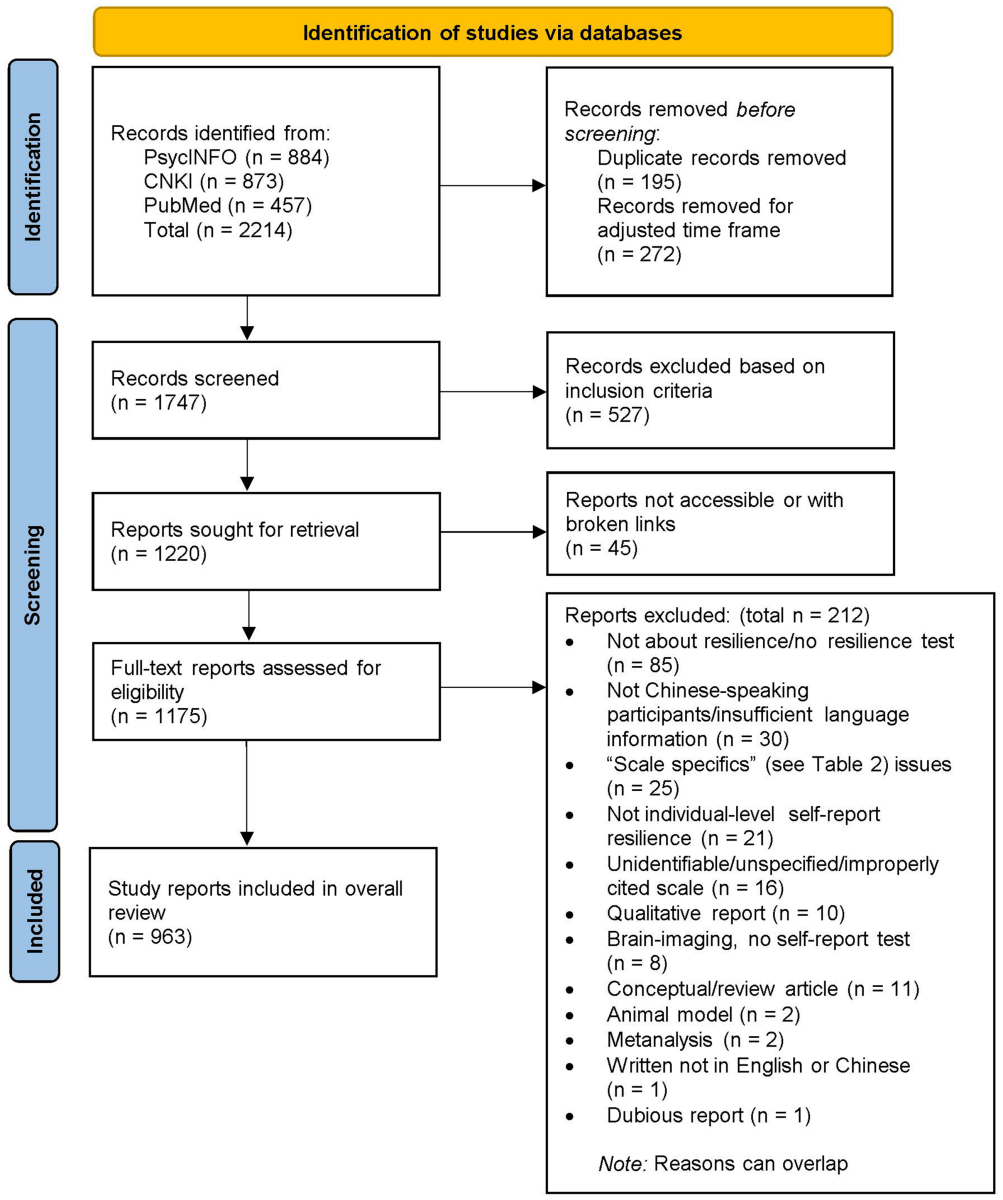
The review process (adapted from Page et al., 2021; the PRISMA template is distributed under the terms of the Creative Commons Attribution License).

The first author independently started the screening while the team met periodically to discuss results and ambiguous cases until the completion of a detailed code book (see Table 2). Then, the fourth author screened 50 randomly selected records for inclusion/exclusion to check reliability; however, several disagreements between the two coders occurred (Krippendorff’s alpha = 0.57).^5^ We, therefore, met to further clarify the criteria, after which another interrater reliability check of 50 more studies was run (Krippendorff’s alpha = 0.96), with the sole disagreement addressed through discussion. The first author reexamined coded records and proceeded to screen the remaining, including CNKI and PubMed records. Duplicates were removed.^6^ Assisted by other authors, the first and second authors retrieved full-text reports that passed abstract screening for further assessment; ineligible and inaccessible reports were excluded. Figure 1 demonstrates the screening processes.

### 3.4 Data extraction

#### 3.4.1 Coding study context

To address our first goal, we coded the specific context in which resilience was investigated in each study. Determining context was an emic sensemaking process (i.e., identifying and building categories from the ground up) whereby we identified and coded context categories based on included studies. Detailed instructions and specific categories and descriptions are elaborated in the Supplementary Table S1. For the PsycINFO reports, the team met periodically (e.g., after every 50 studies were coded) to discuss study contexts until the finalization of a context codebook, using which two authors independently coded the CNKI reports (Krippendorff’s alpha = 0.91), while one author coded the later added PubMed reports. Disagreements/uncertainties were addressed through group discussion.

#### 3.4.2 Scale identification and evaluation

The first author recorded the resilience scale(s) used in each report, which was later checked by two other authors. Reports including results about two resilience scales (e.g., CD-RISC and RS) were listed two times, and different versions of the same scale (e.g., CD-RISC-25 vs. CD-RISC-10) were noted. The frequency of each scale became evident through this process, whereby we determined specific scales to be further evaluated. We then referred to in-text citations and references of relevant reports to identify the scale development and/or translation and validation study/studies reporting the original scale development and/or translation work (hereto referenced as “original reports”) for selected scales. If a translated scale had multiple referenced sources of translations in relevant studies, the most frequently referenced translation was selected. For example, for the 14-item Resilience Scale, which had three referenced translated versions, the version by Tian and Hong (2013) was used because it has been used more frequently than others (e.g., Chung et al., 2020). Next, the first and second authors retrieved the original reports for each scale to be evaluated.

Table 1 presents the rubric adapted from Windle et al. (2011), which shows seven criteria (e.g., content validity, internal consistency) relevant to evaluating the psychometric properties of resilience measures in this project, including “factor structure” and “contextualized translation” that were added given their importance for assessing scales across languages and cultures (Farh et al., 2006). The rubric follows Windle et al.’s (2011) 3-point scoring system, including “2” fully meeting, “1” partially meeting, and “0” failing to meet (or missing information about) a given criterion. For example, for internal consistency, a “2” rating means that factor analyses with adequately sized samples (i.e., 7* #items and > = 100) have been conducted and Cronbach’s α values 0.70–0.95 per dimension have been reported (Terwee et al., 2007). A “1” can mean doubtful design (e.g., inadequate sample size) or, for a multidimensional scale, Cronbach’s alphas for no more than half of its dimensions are outside of the previous range. Finally, a “0” means problematic value(s) per dimension regardless of design and/or missing information (for more details, see Table 1). Using Windle et al. (2011) to further illustrate, regarding internal consistency, a scale receives a “1” for not reporting Cronbach’s alphas for subscales despite acceptable whole-scale alpha, whereas a one-factor (tested through EFA and CFA) scale with an alpha between 0.70 and 0.95 gets a “2.” For test–retest reliability, a scale is rated “1” despite good ICC (e.g., 0.87) for inadequate retest sample size (<50), whereas a “0” is given to a scale without reported test–retest reliability. Importantly, the scores are ordinal, meaning that a score for one specific criterion enables the ranking of selected scales by the given criterion; sum scores per scale enable a ranking of scales on overall psychometric qualities (with seven criteria, sum scores for measures can range from 0 to 14 points, with 14 being the highest possible score). Terwee et al.’s (2007) system uses symbols (e.g., “+”/“?”), which is less effective in demonstrating overall qualities.

Using the criteria, the two authors evaluated original reports and corresponding scales independently. The team met to clarify the rubric and address disagreements. Then, one author performed the rating independently, and all three coders reached 100% agreement. Although most evaluation criteria were assessed from the original reports, we sought information about some criteria (e.g., internal consistency and test–retest reliability) from relevant articles. Additionally, two authors recorded Cronbach’s alphas of the evaluated scales reported in relevant reports (if available). For scales used over 100 times (CD-RISC and RSCA), we randomly selected 50 studies for each scale.

## 4 Results

In total, 963 reports (301 PsycINFO, 551 CNKI, 111 PubMed) met the inclusion criteria and were included in this review (see Figure 1). In these included articles, resilience assessment occurred 973 times in various contexts using a range of measures (some studies used two to three resilience scales, e.g., Li et al., 2018). Among these assessments, we identified 85 unique self-report resilience measurement scales (a scale and its variations, such as short forms, are considered one in this count) (see Supplementary Table S2).

### 4.1 Chinese resilience research contexts

Our first aim was to identify contexts where resilience testing has been conducted. Among the 963 research reports, the largest group (*n* = 332, 34.5%) focused on *health* conditions. These studies were characterized by their clear foci on mental or physical disorders (e.g., schizophrenia and bipolar disorder; Deng et al., 2018), diseases (e.g., HIV/AIDS; Gao et al., 2018), illnesses (e.g., cancer; Ye et al., 2018), and/or public health concerns (e.g., aging framed in such a matter; Tan et al., 2016). This group also included studies on temporary or lasting conditions where some level of medical care was required (e.g., pregnancy; Ma et al., 2020). In these studies, participants were commonly patients, survivors, or caregivers.

Next, 176 studies (18.3%) used resilience scales in a *general* context as part of a survey for a mental health index/profile of a population not known for facing specific risks, such as “healthy individuals” and college students in some cities (e.g., Kong et al., 2018). The general context usually considered no specific stressor, or alternatively, a range of adversities/stressors/risk factors such as unspecified childhood adversity (Li et al., 2014) or chronic or short-term stress as a common experience (Ramsay et al., 2015; Shi and Wu, 2020). Two contexts determined by the physical settings (of social organizing) were *work*, occupational, and/or organizational challenges (*n* = 158, 16.4%) and *school* life and academic challenges (*n* = 32, 3.3%). Studies in these categories usually focused on routine challenges associated with these settings, such as workplace burnout and fatigue common to stressful occupations (e.g., medical professionals and civil servants; Qiu et al., 2020) and academic burnout (Ying et al., 2016).

The next context concerned experiences and trends associated with systemic, socio-cultural-economic phenomena in and beyond contemporary China (*n* = 158, 16.4%). These studies examined a range of overlapping, publicly aware “*social* problems” as complex forms of adversity, often involving the marginalization of specific populations, such as urban–rural migrant workers (e.g., Yang et al., 2020) and families (e.g., Gao et al., 2020), left-behind children (e.g., Gao et al., 2019), migration/immigration (e.g., Yu et al., 2015), and LGBTQ+ groups (e.g., Yang et al., 2016). Moreover, resilience scales were also used in micro contexts concerning the common and specific challenges of *relating*, including personal, family, and community relationships (*n* = 58, 6.0%), such as parent–child conflict (Tian et al., 2018) and older adults losing their sense of community (Zhang et al., 2017). Additionally, we decided to present *abuse* and bullying as a unique context (*n* = 31, 3.2%), given the specificity of the behaviors, usually with the intention to harm, and the ways such events involve similar experiences of victimization (e.g., fear and isolation) across settings (e.g., school, workplace, and family; Zhou et al., 2017; Lin et al., 2020).

Furthermore, researchers examined resilience in the context of *natural disasters* (*n* = 29, 3.0%), such as post-traumatic stress disorder and growth linked to experiencing an earthquake (Xi et al., 2020) or rainstorm disaster (Quan et al., 2017) and *shidu* after an earthquake (Wang and Xu, 2017).^7^ Finally, 31 studies (3.2%) focused on resilience during the *COVID-19 pandemic* (e.g., Ye et al., 2020). We identified the pandemic as a separate context due to the magnitude of this atypical event and the multiple ways in which it disrupted social order. In sum, these findings show the broad range of life disruptions explored in interdisciplinary resilience research with Chinese populations.

### 4.2 Chinese resilience scales

Our second aim was to identify which resilience scales have been used most frequently with Chinese-speaking populations, including whether the popularity of scales varies across contexts. Only three scales (including their variations) accounted for *at least 5%* of the total 973 times of resilience assessment, including the CD-RISC scales (54.9%), RSCA (12.5%), and various versions of RS combined (8.4%). The CD-RISC and RS were translated scales, whereas the RSCA was a locally developed measure.

Given our desire to focus on popular scales, including both translated and locally developed, we decided to limit our focus to the three most frequently used translated scales (including both the original version and common variations) and a matching number of locally developed scales when conducting a detailed evaluation of psychometric properties (see below). We specifically focused on the following nine Chinese scales organized into six groups: (1) CD-RISC-25 (Yu and Zhang, 2007) and CD-RISC-10 (Wang et al., 2010); (2) RSCA (Hu and Gan, 2008); (3) three versions of the RS including RS-25 (Lei et al., 2012), RS-14 (Tian and Hong, 2013), and RS-11 (Gao et al., 2013); (4) the 14-item version of the Ego-Resiliency Scale (ER-14 CN; Chen et al., 2020); (5) the Essential Resilience Scale (ERS; Chen et al., 2016); and (6) the Resilience Trait Scale for Chinese Adults (RTSCA; Liang and Cheng, 2012). To address which of these scales were used most frequently in different contexts, we performed a cross-tabulation analysis to show how “popular” these scales were in each context. The CD-RISC-25 was the most popular choice across most study contexts, including “general” (49.7%), “health” (67.0%), “work” (68.9%), “school” (41.7%), “relational” (42.0%), “disaster” (79.2%), and “COVID-19” (57.1%). The RSCA was used most frequently in the remaining “systemic” (52.4%) and “abuse” (40.9%) contexts (see Table 3). In short, CD-RISC-25 dominated resilience testing in Chinese.

**Table 3:** Scale * context crosstabulation (only counting reports related to evaluated scales).

			Contexts									Total
			General	Health	Systemic	Work	School	Relational	Natural disaster	COVID-19	Abuse	
Scale	CD-25	Count	72	200	22	71	10	21	19	16	8	438
		% within context	**49.7%**	**67.0%**	26.2%	**68.9%**	**41.7%**	**42.0%**	**79.2%**	**57.1%**	36.4%	**56.4%**
	CD-10	Count	16	42	2	12	2	3	1	11	2	91
		% within context	11.0%	14.1%	2.4%	11.7%	8.3%	6.0%	4.2%	39.3%	9.1%	11.7%
	RSCA	Count	26	12	44	1	8	20	1	0	9	121
		% within context	17.9%	4.0%	**52.4%**	1.0%	33.3%	40.0%	4.2%	0.0%	**40.9%**	15.6%
	RS-25	Count	7	6	4	1	2	3	2	0	1	26
		% within context	4.8%	2.0%	4.8%	1.0%	8.3%	6.0%	8.3%	0.0%	4.5%	3.3%
	RS-14	Count	8	27	3	3	0	0	0	0	0	41
		% within context	5.5%	9.1%	3.6%	2.9%	0.0%	0.0%	0.0%	0.0%	0.0%	5.3%
	RS-11	Count	4	3	0	1	0	1	0	0	1	10
		% within context	2.8%	1.0%	0.0%	1.0%	0.0%	2.0%	0.0%	0.0%	4.5%	1.3%
	ER-14	Count	8	6	6	7	2	1	1	1	1	33
		% within context	5.5%	2.0%	7.1%	6.8%	8.3%	2.0%	4.2%	3.6%	4.5%	4.2%
	RTSCA	Count	0	0	2	6	0	1	0	0	0	9
		% within context	0.0%	0.0%	2.4%	5.8%	0.0%	2.0%	0.0%	0.0%	0.0%	1.2%
	ERS	Count	4	2	1	1	0	0	0	0	0	8
		% within context	2.8%	0.7%	1.2%	1.0%	0.0%	0.0%	0.0%	0.0%	0.0%	1.0%
Total		Count	145	297	84	103	24	50	24	28	22	777

Bold values mark which scale is most frequently used within a given context.

### 4.3 Scale quality

Our third aim was to assess the psychometric properties of widely used translated and locally developed Chinese resilience measures. The nine scales (including multiple variations of the top six scales) were evaluated based on seven criteria. Scales received a score of “0” (e.g., no information provided) to “2” (e.g., criterion met using rigorous procedures) for each criterion, resulting in an overall score ranging from 0 to 14 possible points. Overall, locally developed scales tended to score higher than translated ones, but none achieved the highest possible rating (see Table 1 for the rating system and Table 4 for ratings of each scale).

**Table 4:** Summary of scale assessments.

Measure	Content validity	Internal consistency	Factor structure	Construct validity	Test–retest reliability	Interpretability	Contextualized translation	Total
The 25-item Connor–Davidson Resilience Scale (CD-RISC-25 CN)	? 1	? 1	+ 2	+ 2	- 0	- 0	? 1	7
The 10-item Connor–Davidson Resilience Scale (CD-RISC-10 CN)	? 1	+ 2	+ 2	+ 2	? 1	? 1	? 1	10
The 25-item Resilience Scale (RS-25 CN)	? 1	+ 2	+ 2	? 1	? 1	? 1	? 1	9
The 14-item Resilience Scale (RS-14 CN)	? 1	+ 2	+ 2	? 1	? 1	? 1	? 1	9
The 11-item Resilience Scale (RS-11 CN)	? 1	+ 2	- 0	? 1	- 0	? 1	? 1	6
The Ego Resiliency Scale (ER-14 CN)	? 1	+ 2	+ 2	? 1	- 0	? 1	+ 2	9
Resilience Scale for Chinese Adolescents (RSCA)	+ 2	+ 2	+ 2	+ 2	- 0	? 1	+ 2	11
Essential Resilience Scale (ERS)	+ 2	+ 2	+ 2	+ 2	- 0	? 1	+ 2	11
Resilience Trait Scale for Chinese Adults (RTSCA)	+ 2	? 1	+ 2	? 1	? 1	- 0	+ 2	9

#### 4.3.1 Content validity

The three locally developed measurement scales (i.e., RSCA, ERS, and RTSCA) received the full score for content validity. All three clarified their aims, discussed resilience and its cultural relevance in Chinese contexts, and involved the target population in item creation/selection (e.g., items were written based on themes in interviews with Chinese participants). Although all translated scales clarified aims and defined resilience, descriptions of target population involvement in the item translation/selection process were not found in the original articles reporting the Chinese versions of these scales.

#### 4.3.2 Internal consistency

Regarding the CD-RISC-25, Cronbach’s alpha for one of the subscales (optimism) was only 0.60 (Yu and Zhang, 2007). The same subscale has displayed less than optimal internal consistency in other studies as well (e.g., Cai et al., 2017). For RTSCA, the internal locus of control subscale (out of five) was 0.60. Nonetheless, all studies reported accepted Cronbach’s alphas (i.e., 0.70–0.95) for the total scales with adequate sample sizes. Additionally, we calculated the average alphas (if reported) of these scales. Results were as follows: CD-RISC-25 and –10 = 0.90 and 0.90; RS-25, −14, and −11 = 0.92, 0.90, and 0.86; ER-14 = 0.82; RSCA = 0.83; ERS = 0.92; RTSCA = 0.86.

#### 4.3.3 Factor structure

The authors of RS-25, ER-14, and RSCA examined the proposed or revised factor structures of their measures by conducting EFA and CFA in separate samples. Additionally, for the CD-RISC-25, CFA results failed to retain the original five-factor structure; therefore, Yu and Zhang (2007) conducted EFA and proposed a three-factor structure for the scale. Although they did not replicate the three-factor structure in an independent sample, other researchers have done so (e.g., Xie et al., 2016). Similarly, ERS’s three-factor structure tested through a single CFA (Chen et al., 2016) was replicated by Lau et al. (2020). RTSCA’s five-factor structure was confirmed in one sample (Liang and Cheng, 2012) and was replicated in Li et al. (2017). Wang et al. (2010) conducted an EFA to explore CD-RISC-10’s one-factor structure, which was then verified by Cheng et al. (2020) through CFA and in the case of RS-14, Tian and Hong (2013) reported two factors through EFA, which were verified in later structural equation modeling (SEM) analyses (e.g., Huang et al., 2020). Therefore, these measures were rated highest (i.e., “2”) for the factor structure criterion. RS-11 received a “0” because insufficient information was available to judge whether its factor structure was supported or replicated.

#### 4.3.4 Construct validity

Four measures (CD-RISC-25 and -10; RSCA; ERS) achieved the full score regarding construct validity. The other scales received the intermediate score due to a lack of clarity. That is, although validation studies for measures assessed resilience along with other literature-informed, conceptually related constructs and reported significant relationships among them, the authors did not formulate hypotheses or clearly state some type of expected relationship (directional or not) between resilience and these constructs before reporting statistical tests.

#### 4.3.5 Test–retest reliability

Adequate information about test–retest reliability was available for five measures (CD-RISC-10, RS-25, −14, and −110, and RTSCA) in their original or most popular versions. However, none achieved the full score for two reasons: failing to report intraclass correlation coefficients and using small sample sizes. The test–retest correlation coefficient for CD-RISC-10 was 0.90 across two weeks, 0.31 after six months for RS-25, and “ranged from 0.53 to 0.85” with “86% > 0.70” for RS-14 (Tian and Hong, 2013, p, 1500). The sample sizes for these studies, however, were small (below 40), which constitutes a design issue according to Windle et al. (2011), given that correlations from small samples contain greater sampling error. For RTSCA, the coefficient for the full scale was 0.88 after three weeks; however, the coefficients for three of the five dimensions were smaller than 0.70; its retest sample of 47 individuals also did not fully meet the criterion. The coefficient for RS-11 was 0.62, therefore failing to meet the criterion. The authors of RSCA (Hu and Gan, 2008) mentioned a “retest” in Chinese; however, it used a new group of participants instead of returning members of an existing sample. It is worth noting that although test–retest information was missing from Yu and Zhang (2007), one included study (Xie et al., 2016) that examined the psychometrics of this version of CD-RSIC-25 did report test–retest reliability of 0.66 across two months. The coefficient for a version of Chinese ER-14 utilized in an unpublished dissertation (cited by a few; Li, 2008) was 0.71 across a month with a retest sample of 198 people.

#### 4.3.6 Interpretability

Information demonstrating potential differences in scoring between or among subgroups of a reference population was available for all but CD-RISC-25 and RTSCA. However, none achieved the maximum score because they did not identify and report results about at least four subgroups and/or present the means and standard deviations.

#### 4.3.7 Contextualized translation

Four measures (ER-14, RSCA, ERS, and RTSCA) fully met this added criterion that considered whether researchers (a) clarified/justified whether they viewed resilience as universal, context-specific, or containing elements of both and (b) considered cultural and linguistic appropriateness during scale development or translation. RSCA was perhaps the most rigorous as Hu and Gan (2008) considered different available translations for resilience and chose the most suited one by referencing Taoist values and then creating items informed by the results of thematic analysis of qualitative interviews. For the ERS, Chen et al. (2016) considered resilience a universal concept, and they developed and revised the wording of scale items in both Chinese and English by consulting experts and conducting pilot tests with native speakers in both contexts. For the RTSCA, Liang and Cheng (2012) explained available translations for “resilience” emphasized “Chinese cultural background” when interviewing experts for item generation and conducted a pilot test to finalize items. When Chen et al. (2020) translated the ER-14 CN, they established their position (universal and cultural-specific) and addressed how items may have changed in different translations. The translation and back-translation were an iterative process involving researchers, a third-party expert fluent in both languages, and another reviewer. Other translated scales used the standard translation-back-translation technique without additional information on contextualized translation, therefore receiving the intermediate score.

## 5 Discussion

Scholars have stressed the importance of cultural and social contexts for understanding the shapes, trajectories, and determinants of resilience (Southwick et al., 2014). Our review identified nine such contexts in which empirical testing of resilience was conducted (e.g., health/illness, natural disasters, workplace challenges). After identifying 85 groups of unique self-report scales, we chose to focus on three widely used translated scales and three locally developed scales, which, when different versions of the same measure were considered (e.g., CD-RISC-25 and CD-RISC-10), resulted in nine total scales. The CD-RISC scales accounted for over half of the reported tests in our sample, and perhaps not surprisingly, CD-RISC-25 was the most popular measure in general, health, work, school, relational, disaster, and COVID-19 contexts. Considering the CD-RISC-25’s immense popularity and contribution, we discuss how its application might be advanced. Following this, we offer recommendations for future resilience scale development/refinement, including more focus on process-oriented scales, and acknowledge the limitations of our systematic review.

### 5.1 Future considerations for CD-RISC-25-CN

Extant knowledge about resilience in Chinese contexts relies heavily on the translated CD-RISC-25, yet the scale scored only moderately (7/14) on overall quality (see Table 4). Given this, future research should continue scrutinizing the scale’s content validity and translation. The simultaneous universality and cultural specificity of resilience have been widely acknowledged, which Yu and Zhang (2007) discussed when explaining the changed factor structure for their translation of the CD-RISC-25 (i.e., three factors in Chinese contexts as opposed to five factors in U.S. contexts). Since their original contribution, however, researchers have not further explored whether the scale taps qualities that may be saliently associated with resilience in Chinese contexts. For example, the items in both languages are notably individualistic (e.g., I will/can/take the lead), while “traditional” Chinese cultures are characterized by communal and relational orientations and the contemporary Chinese “self” is socially and individually oriented (reflecting the merger of global cultures; Kolstad and Gjesvik, 2014), what Lu and Yang (2006) termed a “composite self.” Given this, research might assess whether the scale’s content, predictive, and convergent validity might be enhanced by modifying or adding items (Farh et al., 2006) to tap these qualities. As a second example, Yu and Zhang (2007) stated that “Chinese people are probably the least religious people in the world” (p. 27) to explain the changed factor structure and no longer salient “spiritual influence” with one item explicitly mentioning “God” merged into “optimism” (which might in part explain why the alpha value of this specific dimension has been subpar across studies). Resilience researchers might consider how Chinese contexts are characterized by religious/spiritual diversity (rather than simply lacking religion; Chao and Yang, 2018). These issues could be addressed by reexamining the content validity and language of the current version and subsequently updating the scale, whose factor structure could then be explored and test–retest reliability reexamined. Our point here is not to discount the value of research findings based on the translated CD-RISC-25, as the scale clearly has been heuristic. We suggest that scale validation is an ongoing process, and issues such as content validity and contextualized translation are critical to consider when more than half of recent Chinese resilience studies have employed this measure. Advancing this influential scale is one critical area for future research.

It is worth noting that RSCA and ERS, both developed in China and involving Chinese-speaking populations, scored the highest (11/14 points). Given that the RSCA focuses on adolescents, one direction for researchers is to develop a version for the general population through similar rigorous processes (see Hu and Gan, 2008; discussed more later). The ERS, which is relatively new, needs further validation across contexts. Importantly, test–retest reliability for both RSCA and ERS is yet to be established.

### 5.2 Future recommendations

In this section, we draw on evaluation results to provide seven methodological recommendations for future resilience scale development work with existing and new scales worth considering in and beyond the Chinese study contexts.

*First,* regarding content validity (see Section 4.3.1 and Table 1), *future work could explicitly engage with target populations and/or consult third-party experts* (e.g., someone who is familiar with resilience in Chinese contexts either because of lived experience or extensive learning) in item translation, selection, and/or adaptation. These additional processes, which may result in modified item wording, are commonly expected in new-scale development (Worthington and Whittaker, 2006). Developing culturally adapted versions of existing scales in a new language should not be exempted from these steps (Farh et al., 2006).

*Second, studies should habitually report Cronbach’s alphas for scales, and, for multiple-dimension instruments, consider evaluating internal consistency for subscales rather than only overall scales.* These practices were surprisingly absent in many of the included studies. In addition, scholars recently have argued that McDonald’s omega is a better test of a (sub)scale’s internal consistency (i.e., unidimensionality), so future research should consider reporting omega (Goodboy and Martin, 2020; Hayes and Coutts, 2020).

*Third, future work should carefully examine the contextual translation of resilience scales (see* Farh et al., 2006*) as well as their factor structure in unique cultural situations.* We added a new criterion to evaluate the factor structure of a scale. As shown in existing work, such as the case of CD-RISC-25 (Yu and Zhang, 2007), the factor structure can be sensitive to translation (Chen et al., 2020). When introducing, translating, and applying a scale developed and validated in a different source language and cultural context, researchers must consider the heterogeneity regarding the factor structure of translated versions. Specifically, variations in how items are translated may result in inconsistency. For example, although Tian and Hong (2013) reported a two-factor structure for RS-14—which was replicated in other studies in both simplified and traditional Chinese (e.g., Chung et al., 2020)—a new translation of the scale showed a one-factor structure (Chen et al., 2020). Moreover, translating a generic scale into a more specific context (or vice versa) in the same language may also yield a changed factor structure. For example, when Hao et al. (2015) adapted the five-factor RTSCA for the specific occupation of civil servants, EFA suggested a four-factor structure instead.

*Fourth,* given that more than half of the evaluated scales were tested without clear hypotheses regarding resilience’s relationship with study constructs (i.e., dubious design, Windle, 2010), *future work with new or existing scales should clearly articulate their rationale for testing associations between resilience and associated constructs as informed by theory and/or existing literature, rather than only mentioning possible relationships among constructs.*

*Fifth, future researchers should include test–retest with adequate sample sizes and justified time intervals in their design.* Test*–*retest reliability is the most problematic property in the results (see Table 4). When assessed at all, researchers tended to perform retests using small (below 50) samples, which may partly explain the dubious coefficients (below 0.70) reported in some studies.^8^ Test–retest results could further inform a discussion on situations under which resilience should be expected to remain stable or change over time. For example, a trait measure that assumes the stability of resilience over time should result in higher test*–*retest coefficients than a measure based on the changing process view on resilience, where the way in which resilience is enacted might change over time (see below). Additionally, only a few studies reported the ICC (e.g., Lo et al., 2014; Hsieh et al., 2016) even though Terwee et al. (2007) deemed it “the most suitable and most commonly used reliability parameter for continuous measures” for its consideration of “systematic differences [as] part of the measurement error” (p. 37). Future studies should report the ICC.

*Sixth, future work could begin exploring minimal important change (MIC), concerning interpretability, for Chinese resilience scales*. MIC was not assessed for selected scales (as it was not for English scales, see Windle et al., 2011), which was indeed acknowledged as a limitation in an excluded Singaporean study, where CD-RISC-10 was validated in English-speaking patients with axial spondylarthritis (Kwan et al., 2019). In clinical research, MIC concerns the threshold where patients begin to perceive their internal change over the course of treatment to be important, which enriches the interpretation of results from the perspective of recipients of the treatment (for a systematic guideline on methods, see Terwee et al., 2021). Exploring MIC in future research could promote an understanding of resilience stemming from dialogue between researchers and participants (e.g., through anchor questions; Terwee et al., 2021), rather than based on assumptions that small but statistically significant changes in researcher-defined outcomes would have a meaningful impact on participants’ lives.

*Seventh, future researchers should address the universality and/or cultural specificity of their concept of interest as well as consider how similar (but nuanced) experiences and phenomena are presented across specific living languages* (see Farh et al., 2006). This suggestion reflects our alignment with the current consensus that considers resilience as both “universally observable” and “culturally specific” human (biological and social) experiences expressed in numerous ways (Southwick et al., 2014). Regardless of whether scholars translate an existing scale from another culture or locally develop a scale for self-report instruments, procedures for ensuring that the items make sense to a variety of participants in the target language could further enhance rigor and ethics, as well as contribute to content validity. Translation work requires taking additional steps beyond standard translation and back-translation. A recent effort in translating, applying, and validating the 14-item ego resiliency scale (Chen and Padilla, 2019; Chen et al., 2020) presents an example of researchers taking manageable steps to demonstrate awareness and sensitivity to culture and language. Chen and colleagues considered resilience to be both universal and culturally specific and addressed how the scale items had changed in previous translation studies. Their back-translation involved several experts fluent in both English and Chinese, who met to reach a conceptual and translational consensus. The version was then reviewed by a third party. Researchers might also gather pilot data from both expert and lay persons and adjust the translation accordingly, similar to early-scale development (Worthington and Whittaker, 2006).

### 5.3 Moving beyond trait conceptions of resilience

We also call for more future attention on developing and assessing the psychometric qualities of process-oriented measures in individual resilience in Chinese-speaking contexts. Scholars in our review predominantly took the trait approach to resilience assessment in that over half of the tests employed some version of the CD-RISC scale, which is known for its trait view. Original authors of all but one evaluated scale also aligned with the trait view. For example, for the RS-25, resilience is defined as a “personality characteristic that moderates the negative effects of stress and promotes adaptation” (Wagnild and Young, 1993, p. 165). Ego-resilience/resiliency, as the name implies, is considered part of the ego structures maintaining the personality system (Block and Kremen, 1996). Notwithstanding the importance of examining how resilient traits or trait resilience relate to other phenomena, interdisciplinary resilience theorizing has endorsed a more complex and comprehensive process views where resilience systems consider interconnected mechanisms mobilizing protective and risk factors (Windle, 2010; Panter-Brick and Leckman, 2013; Southwick et al., 2014). Luthar et al. (2000) have long suggested that examining what (and to what extent) specific mechanisms (e.g., informal support) mediate the effect of protective factors (e.g., religiosity) is crucial for prevention and intervention designs for populations in need (e.g., Chiu et al., 2020). In short, individual-level resilience assessment in Chinese contexts in the recent past has not moved very far from the long-established individual-trait view; available tools from process perspectives remain mostly underused or underdeveloped in translation work. To encourage resilience process assessment in Chinese contexts, we help highlight some process-focused measures and discuss future directions.

Two of the more frequently used scales (see Supplementary Table S2) are based on process views of resilience. The RSCA (Hu and Gan, 2008) attempts to assess resilience as a dynamic process through which adverse life events interact with protective factors. Nevertheless, specifically developed from the perspective of adolescents and largely concerning the parent–child relationship, this scale may not be appropriate for other contexts and populations (e.g., adults experiencing chronic illness). Hu and Gan demonstrated a rigorous way of involving target populations and developing a scale from the ground up before testing and revising new samples. Studies replicating their procedures (and adding testing–retesting reliability) in adult and/or general samples to develop population/context-appropriate scales could advance the current resilience process studies. In addition, the Resilience Scale for Adults (RSA) conceptualizes resilience as a multidimensional construct referring to “important psychological skills or abilities [and] the individual’s ability to use family, social and external support systems to cope better with stress” (Friborg et al., 2003, p. 66), which aligns with seeing resilience as complex processes encompassing interacting protective systems (e.g., Rutter, 1990). However, the English-to-Chinese translation needs further scrutiny and development, considering that there are several referenced translations of RSA and that the factor structures of the scale were not consistent across different samples (e.g., Yang and Lv, 2008; Peng et al., 2011; Ma et al., 2019).

Furthermore, process views may focus on the connections between individuals and surrounding systems, including how protection/adaptation emerges from the interactive process (Masten, 2015). For example, another translated, population-specific scale in our data, the Child and Youth Resilience Measure (CYRM; Liebenberg et al., 2012, 2013; Ungar and Liebenberg, 2011) operationalizes resilience process with emphases on specific communicative practices (e.g., talking about adversity itself), social construction, nonstatic interpretation, and contextual sensitivity. Although not yet used often, Xiang et al. (2012) translated and performed factor analyses for the 28-item version, which resulted in a 27-item Chinese version. Early translation and validation work of the 12-item version has been provided by Mu and Hu (2016). The Family Resilience Assessment Scale FRAS (Sixbey, 2005) based on Walsh’s (2016) family resilience framework is worth mentioning for similarly tapping family interactions, though the FRAS has at least three Chinese versions with different numbers of items (see Li et al., 2016; Fan et al., 2017; Chiu et al., 2019). More recently, Wang and Lu (2022) translated and initially validated Walsh’s (2016) questionnaire. Although we have excluded these latter scales from this review, given our focus on self-report measures for *individual-level* resilience, these scales should be of interest to family resilience researchers who wish to advance process views on resilience.

Additionally, we highlight a recent development in resilience theorizing that fully commits to the focus on social interaction. Buzzanell (2010, 2019) has proposed a communication theory of resilience (CTR) that theorized resilience as five communication processes, which different levels of agentic actors (e.g., individuals and organizations) perform/enact to maintain and/or transform normalcy and meaningfulness in responses to disruptive trigger events.^9^ Based on this theory, Wilson et al. (2021) developed a 32-item Communication Resilience Process Scale (CRPS) in a series of three studies with participants from the United States. The CRPS includes seven subscales tapping the five CTR resilience processes at the individual level (e.g., the process of “crafting normalcy” is subdivided into maintaining current routines and creating new ones). When using the CPRS, researchers first ask participants to recall and describe a significant life disruption (one event or a series of events) that they have faced within a timeframe (e.g., the past two years) before completing self-report items about the extent to which they *enacted* communicative practices reflective of the resilience processes. Kuang et al. (2022, 2023) recently translated and contextualized the CRPS into a Chinese version with a retained factor structure but six additional items and slight changes to item wording based on pilot results and feedback from native speakers and experts. The validation work of any measurement is, of course, an ongoing process. Therefore, this process scale needs to be further evaluated in terms of a full range of psychometric standards.

### 5.4 Limitations

Resilience scholarship is growing quickly; therefore, while capturing trends in the recent past, we are simultaneously missing new ones. A quick search in any database would show that resilience publications had continued growing since 2021, likely due to the pandemic. Researchers should consider conducting similar reviews in future to track ongoing attempts to develop and validate resilience measures. By the same token, scholarship on resilience would also benefit from similar measurement reviews for other languages and cultures. Next, multiple translations of “resilience” are used in Chinese studies, which may orient the conceptualization of resilience differently (Hu and Gan, 2008). We did not take into consideration the various translations in the screening and coding for this review; hence, this task could be addressed in future work. Similarly, how well a scale fits its target population age-wise is also an important future concern. Furthermore, our data and screening results might be limited due to not using chain referential sampling to find all possible existing studies and unique scales; however, the 963 reports we identified are sufficient to capture trends such as which research contexts are being explored and which scales are being used most often (i.e., the nine scales we evaluate in depth). Finally, considering the scope and limited available resources, we only used CNKI to collect reports published in Chinese. Future reviews should consider including other Chinese information sources as well.

In closing, we present this first effort to systematically review Chinese resilience measurement scales, in which we identify a list of contexts relevant to empirical testing of resilience in Chinese and commonly used scales. Based on these results, we direct researchers’ attention to actions and practices through which future instrument development work can be more rigorous, such as when assessing test–retest reliability or translating English scales for different languages and populations. Because studies have predominantly provided validation evidence for scales based on trait conceptualizations of resilience, we also call for more focus on developing/evaluating process measures that assess how Chinese individuals and groups create or enact resilience in response to life’s inevitable disruptions.

## Data availability statement

The original contributions presented in the study are included in the article/Supplementary material, further inquiries can be directed to the corresponding author.

## Author contributions

ZT: Writing – review & editing, Writing – original draft, Visualization, Validation, Supervision, Resources, Project administration, Methodology, Investigation, Funding acquisition, Formal analysis, Data curation, Conceptualization. KK: Writing – review & editing, Visualization, Validation, Supervision, Project administration, Methodology, Investigation, Formal analysis, Data curation, Conceptualization. SW: Writing – review & editing, Validation, Supervision, Methodology, Investigation, Formal analysis, Conceptualization. PB: Writing – review & editing, Validation, Supervision, Methodology, Investigation, Formal analysis, Conceptualization. JY: Writing – review & editing, Formal analysis, Data curation. XM: Writing – review & editing, Formal analysis, Data curation. HW: Writing – review & editing, Formal analysis.

## Footnotes

^1^It is important to clarify that in this review, we aim at the broad, plural sense of Chinese contexts, by which we refer to the use of Chinese (as a language group) and cultural affiliations rooted in historicity.

^2^Terwee et al.’s (2007) proposed psychometric properties include as follows: (1) content validity, (2) internal consistency, (3) criterion validity, (4) construct validity, (5) reproducibility (including agreement and reliability), (6) responsiveness, (7) floor and ceiling effects, and (8) interpretability. However, several criteria (i.e., gold standard, reproducibility-agreement, floor and ceiling effects, and responsiveness) are typically meaningful in medical/clinical contexts (e.g., clinical trials, interventions, and changes) but are less relevant to the current stage of resilience research (see Windle et al., 2011; Zhou et al., 2020; Windle et al., 2022) and thus were excluded from this review.

^3^Zhou et al. (2020) systematically reviewed *family resilience* questionnaires in both English and Chinese studies; however, our manuscript focuses on individual-level measurement, given that most Chinese resilience scales are cast at the individual level. Only considering English reports, Windle et al.’s (2022) review is specific to scales for people (samples from various countries) living with dementia and their caregivers and hence is less relevant to our review, which looks at scales for Chinese-speaking populations who are facing a wide range of disruptive life events.

^4^The data were collected from contexts where Chinese was the primary language, regardless of whether the research report was written in English or Chinese. These criteria enabled us to infer the primary research language if not explicitly identified. For example, a study whose sample was a local community in Shandong province or Hong Kong most likely used scales in simplified or traditional written Chinese (syntactically and semantically similar if not identical), whereas a study whose participants were South African women was unlikely to have used Chinese. Based on the screening of PsycINFO articles, we decided to focus on studies utilizing Mainland Chinese, Hong Kong, Macao, and/or Taiwanese samples because all qualified articles’ samples were affiliated with one or more than one of these areas/regions. Articles were excluded, if the information provided was inadequate to infer the use of Chinese resilience scale(s) (e.g., no mention of the study population).

^5^Krippendorf’s alpha was calculated using Hayes and Krippendorff’s (2007) SPSS macro and selecting nominal as the level of measurement, given that whether a study should be included (yes/no) is a nominal-level variable.

^6^There were 193 duplicate records between PsycINFO and PubMed, two between PubMed and CNKI, and none between PsycINFO and CNKI. We used a Chinese search query in CNKI, which enabled us to include many reports published in Chinese.

^7^These three examples also illuminated other contexts; however, as acute, highly disruptive events that overturn not only individual lives but also environments, natural disasters could override other more routine challenges.

^8^The size of test–retest result depends on (a) the time interval between tests (longer intervals may justify smaller correlations) and (b) sample size (small samples have more sampling error).

^9^Specifically, people adapt and/or transform by *crafting normalcy* (e.g., holding onto rituals and creating new routines), *performing identity anchors* (e.g., meaningful roles and values that guide action), *mobilizing communication networks* (e.g., reaching out to strong/weak ties), *enacting alternative logics* (e.g., reframing events, using humor to lighten challenges), and *foregrounding productive action while legitimating negative emotions* (e.g., validating fear/anger while still choosing to take steps toward important goals).
