# Supplementary material for: Measuring resilience for Chinese-speaking populations: a systematic review of Chinese resilience scales
Source: Front Psychol. 2024 Mar 28;15:1293857. doi: 10.3389/fpsyg.2024.1293857 (PMC11007233; doi:10.3389/fpsyg.2024.1293857)
Supplement: Supplementary file 1 [file Table_1.docx]

**Supplemental Table 1: Context Coding Codebook**

| ***Contexts for resilience assessment*** refer to something in/about a study that makes the use of resilience measurement sensible and in general gives a sense of what a study is about. A study’s introduction, and even the title, usually can help identify what is the main issue/topic of the study. Consider the following three things about a study to determine the context. They are NOT mutually exclusive, for example, a setting itself can be a stressor, and a stressor can be a measurable factor. If, with all things considered, a study can still plausibly fit into >= 2 categories, see what issue/problem/event/phenomenon is established the earliest (i.e., primacy) in the introduction.   1. **Stressor**: Resilience is often examined in relation to some type of short-term and/or long-term adversities, difficulties, or challenges (i.e., stressors). Stressors vary in terms of scale and level of complexity. Some are individually experienced conditions or events (e.g., chronic back pain; work burnout), while some others refer to collectively experienced, compounded social events/issues (e.g., rural-urban migration; left-behind children; natural disaster).   *Note:* When a study identifies multiple stressors (e.g., divorce and immigration), consider the primary one or the most salient one. If an order of saliency cannot be discerned, a study likely has a “general context.”   1. **Variables**: Resilience is also often studied in different types of relationships with variables (in terms of traits, states, behaviors, etc.) that are not by themselves stressors but are risk (and protective) factors or outcomes related to these. When a specific variable is the focus (e.g., perceived stress) of a study in which resilience is also tested, context may be determined by it. For example, a study focused on post-traumatic growth (outcome) and predictors, in which resilience is one of the predictors, should be considered health-related (category 2) because the variable of focus is about mental health despite lacking identified stressor(s). 2. **Setting:** Setting also helps determine a context. Many studies focus on stressors and/or factors *specific to* socially recognized settings for interaction (e.g., school, workplace, romantic relationships). In this case, the setting may be what determines the context code. | | |
| --- | --- | --- |
| **Groups** | **Description** | **Examples** |
| 1=General | Studies that study many kinds of or unspecified stressors, do not focus on specific variables, and are in no particular settings.  Resilience assessment can be used along with other mental health variables (e.g., stress, anxiety, depression) to assess the mental health profile or general well-being of a *population that is not in specified health, economic, or other risks*, nor is the population associated with the following more specified categories. | - Mental health index of young people in a city - Unspecified “negative life events” - Unspecified childhood trauma (not attributed to specific cause/source, addressing a variety of outcomes)   *Note*: A study that focuses on childhood trauma and depressive symptoms belongs in health (2) because it focuses on mental health conditions. |
| 2=Health and Medical Conditions  *Note*: Including caregivers’ burden and impacts on significant others (e.g., immediate family members) | Studies that focus on a specific disorder, disease, or illness and/or more general public health concerns (e.g., change in health associated with aging, substance abuse), in the sense that study participants are patients, survivors, a population at risk, or caregivers.  Studies that assess mental health (e.g., stress, anxiety, depression) as outcomes but are focused on specific stressors (e.g., abusive relationships; natural disasters) should be categorized into specific categories. However, if the condition itself is the focus (e.g., a study exploring the relationship between resilience and PTSD), a study goes here. Studies using mental health to assess the baseline of a general population (e.g., the youth of city A) while addressing no specific risk are considered “general.” | - Specific disease or illness (e.g., cancer, diabetes, infertility, chronic back pain, schizophrenia, clinical depression, prenatal anxiety, PTSD, depression suicide ideation) - Taking care of a family member with a chronic illness - Living with HIV/AIDS family members |
| 3=Systemic Socio-Cultural-Economic Issues  *Note*: Must concern social identification, stratification, and mobility (e.g., class/national/international) | Studies that are concerned with systemic, ongoing social events *publicly known in China* (e.g., rural-urban migration) and their impacts on certain populations identified by their association with these issues (e.g., migrant workers). These studies give a sense that the events are the cause of challenging lives. When overlapped with “health,” this category should take precedence because of how much such life experiences systematically impact individual lives.  Compounded with a range of concerns that can be economic and/or cultural, these systemic issues often negatively impact the lives of the populations, manifesting class struggles.  A subject matter just framed as “social” or “global” (e.g., aging population as a social problem) does not immediately belong in this category. Instead, the content must fit the above description. | - Poverty - Rural-urban migration - Left-behind children/adolescents/elders - Immigration - Marginalization |
| 4=Occupational/Work/Organizational Challenges | Studies that concern the professional experience and work-life of workers, employees, or specific professionals. | - Work burn-out of doctors and nurses - Workplace fatigue of banking employees - Training stress of athletes - Military stressors not during wartime (training, heavy workloads, family separation) - Violence healthcare workers may experience due to conflict between them and patients |
| 5=School Life and Academic Challenges | Studies that concern the school life and academic experience of different grades of students. The variables or study background must be specific to the context of school life, such as academic performance and teacher and peer support.  *Note:* Studies using students as the reference population to instead study phenomena not limited to school (e.g., left-behind children; earthquake and PTG) are coded otherwise. Studies using school students used as a reference population that provides normative data for categories such as adolescents or youth are not necessarily category 5. | - Student burnout - Test/exam anxiety - School engagement - College entrance examination - Minority students in Han-dominated schools - Students with disabilities |
| 6=Relational Lives Complexity  *Note:* concerns personal, family, and community relationships, including online relationships | Studies that mainly explore the relationship between resilience and challenges and/or benefits stemming from the complexity of navigating interpersonal relationships.  In terms of family resilience studies, it depends on whether family resilience itself is the focus. For example, a study that assesses the family resilience experienced by cancer patients is coded as “Health, Illness, and Medical conditions.” | - Divorce - Parent-child communication - “Empty-nested elders” (if intergenerational relationships are the focus here) - “*Shidu*” elders (who have lost their only child) |
| 7=Natural Disaster | Studies investigating the difficulties related to experiencing (including anticipating and recovering from) natural disasters. Studies on PTSD and the resilience phenomenon after natural disasters belong to this category. | - Earthquake - Flooding - Landslide - Typhoon/hurricane |
| 8=Covid-19 | Studies that are clearly contextualized by the COVID-19 epidemic/pandemic, such as victimization experience or nurses’ burnout should be categorized as such. | - Covid frontline healthcare worker’s burnout - College students’ experience of COVID |
| 9=Relational Harms | While bullying and abuse are usually experienced through interpersonal relationships at school and work, these risk experiences are present in a variety of settings of interaction and socialization, and therefore this category stands on its own.  Studies that explore the relationship between resilience and experiences of bullying and abuse victimization belong to this category. | - Child abuse and suicide ideation - Ostracism - School bullying - Workplace bullying - Family bullying |
