# Supplementary material for: Measuring resilience for Chinese-speaking populations: a systematic review of Chinese resilience scales
Source: Front Psychol. 2024 Mar 28;15:1293857. doi: 10.3389/fpsyg.2024.1293857 (PMC11007233; doi:10.3389/fpsyg.2024.1293857)
Supplement: Supplementary file 2 [file Table_2.docx]

**Supplemental Table 2: Listing All Unique Measurement Tools for Resilience/y**

| ***Group*** | **Identifying Measurement Tools**  *Note:* Different variations of a scale are put in one group, identified by letters (e.g., a, b, c). Each entry below also links to a sample study (included in our systematic review) *or* a primary scale translation/development and validation report. For a scale originally named in Chinese, if relevant publications identify it in both Chinese and English, we list its Chinese and English names, separated by a slash (“/”). If no English name/translation is provided, we add a literal translation for its name/description in brackets (“[…]”). |
| --- | --- |
| *1* | a. [25-item Conner and Davidson Resilience Scale (CD-RISC-25)](https://doi.org/10.2224/sbp.2007.35.1.19) |
|  | b. [CD-RISC-10](https://doi.org/10.1111/j.1440-1819.2010.02130.x) |
|  | c. [21/23-item CD-RISC for nurses adapted from CD-RISC-25](https://www.cnki.net/KCMS/detail/detail.aspx?dbcode=CJFD&dbname=CJFDLAST2019&filename=SXHZ201911008&uniplatform=OVERSEA&v=HawXvT9xbZD7s_sb_8BmXSP9Y159MYuv0LDYhoW9qShjzg9dF6eQGmQwazq7hRD5) |
|  | d. [CD-RISC-2](https://www.cnki.net/KCMS/detail/detail.aspx?dbcode=CJFD&dbname=CJFDLAST2019&filename=SXHZ201902008&uniplatform=OVERSEA&v=HawXvT9xbZDAlugnq35ijv_8iyv6cMjkGWtmqeTznr3RFfEd-_Rue-J_owH8rUe-) |
| *2* | a. [中国青少年韧性量表/Resilience Scale for Chinese Adolescents (RSCA)](https://journal.psych.ac.cn/xlxb/CN/Y2008/V40/I08/902) |
|  | b. [RSCA adapted for an adult sample](http://dx.doi.org/10.1037/tra0000325) |
| *3* | a. [25-item Resilience Scale (RS-25)](https://doi.org/10.1016/j.comppsych.2011.08.007) |
|  | b. [RS-14](https://link.springer.com/article/10.1007/s00520-012-1699-x) |
|  | c. [RS-11 (German version adapted from RS-14)](https://chn.oversea.cnki.net/KCMS/detail/detail.aspx?dbcode=CJFD&dbname=CJFD2013&filename=JKXL201309024&uniplatform=OVERSEA&v=DHiGUzvR3AN85-waJG5EWvFPCBp1LCksxXecIGGVdj_FeUMIM1pHx7vsvX-IG533) |
|  | d. [RS-15](https://chn.oversea.cnki.net/KCMS/detail/detail.aspx?dbcode=CJFD&dbname=CJFDLAST2020&filename=ZQNZ202002014&uniplatform=OVERSEA&v=aGoazgzjy1JffYQleQyvpcOlFS-a3pYi_oVGUSIPpJdPdxrpHWXWFLascaKijIfJ) |
|  | e. [RS-10 (for Children)](https://doi.org/10.1002/pon.5548) |
| *4* | a. [14-item Ego Resilience Scale (ER-14)](https://doi.org/10.1177/0734282919889242) |
|  | b. [ER-10](https://chn.oversea.cnki.net/KCMS/detail/detail.aspx?dbcode=CJFD&dbname=CJFDLAST2019&filename=XBSW201904013&uniplatform=OVERSEA&v=vRvxkQduNTrXLycrj_Hlux3I8fnVKMaUqYEW5lsMpGza_3RMHOu8j--sypZ2e2uv) |
| *5* | [Resilience Scale for Adults (RSA)](https://doi.org/10.1016/j.jad.2019.02.058) |
| *6* | a. [Psychological Capital Questionnaire (PCQ) 6 resilience items](https://journals.plos.org/plosone/article?id=10.1371/journal.pone.0122128) |
|  | b. [PCQ-12 3 resilience items](https://doi.org/10.1002/hrm.21934) |
| *7* | a. [28-item Child and Youth Resilience Measure (CYRM-28)](https://doi.org/10.1016/j.puhe.2018.05.006) |
|  | b. [CYRM-12](http://dx.doi.org/10.1016/j.childyouth.2016.09.037) |
| *8* | [Essential Resilience Scale (ERS)](https://doi.org/10.1002/smi.2659) |
| *9* | a. [中国成年人心理弹性量表/Resilient Trait Scale for Chinese Adults (RTSCA)](https://chn.oversea.cnki.net/KCMS/detail/detail.aspx?dbcode=CJFD&dbname=CJFD2012&filename=CLXW201204007&uniplatform=OVERSEA&v=y1RQAvHo9wIFeg18f_VqqvDP6D4osFnF1K6CUG4anrO3BqNajS_xLPv0M-JXl9Z8) |
|  | b. [RTSCA adapted for civil servants (with changed factor structure)](https://chn.oversea.cnki.net/KCMS/detail/detail.aspx?dbcode=CJFD&dbname=CJFDLAST2015&filename=ZLCY201505008&uniplatform=OVERSEA&v=jQputrghl3AqjoOlCQLEayBryH67smdhDE-C1SyBZBwHlHxhtj65_lZzt8mQGkNh) |
| *10* | [Brief Resilience Scale (BRS)](https://doi.org/10.1016/j.jadohealth.2020.08.026) |
| *11* | [Chinese Positive Youth Development Scale (CPYDS) resilience subscale](https://doi.org/10.1007/s10964-016-0469-3) |
| *12* | [Resilience Scale Specific for Cancer (RS-SC)](https://chn.oversea.cnki.net/KCMS/detail/detail.aspx?dbcode=CJFD&dbname=CJFDLAST2017&filename=BXYY201705030&uniplatform=OVERSEA&v=0nei9o-YrBpnmCcOSIfSyj24kugs-NF8nAHPA-TWFvw_U7jsG6bbeHSyjKxxgAZn) |
| *13* | [Healthy Kids Resilience Assessment (HKRA)](https://chn.oversea.cnki.net/KCMS/detail/detail.aspx?dbcode=CJFD&dbname=CJFDLAST2017&filename=XLXT201705016&uniplatform=OVERSEA&v=WtvpZ2FBaik1FML3XHk_2tMc8EkP9KwmIhSWJ13igwAfx1iM2XmK4G0SYWJPP-jT) |
| *14* | [Resilience Questionnaire of High School Students (RQHSS)](https://chn.oversea.cnki.net/KCMS/detail/detail.aspx?dbcode=CJFD&dbname=CJFDLAST2018&filename=XDYF201815025&uniplatform=OVERSEA&v=Zpi5ZBLAiQLRH9kHtESXgCppnKxmkwlBd2nJjgu1JARzaE2z7rQrDXj65OWi5IMl) |
| *15* | [中学生心理复原力量表/Self-Rating Scale of Psychological Resilience for Middle School Students](https://chn.oversea.cnki.net/KCMS/detail/detail.aspx?dbcode=CJFD&dbname=CJFD2009&filename=XIWS200912025&uniplatform=OVERSEA&v=K0k1JEiNBAN6w1tZMtQs21Oo4o4Kw4UKZ9UZJ7yqmksqEQ9VIUdbf6vh8hqhHITr) |
| *16* | [积极心理资本问卷/Positive Psychological Capital Questionnaire (PPQ) 7 resilience items](https://chn.oversea.cnki.net/KCMS/detail/detail.aspx?dbcode=CJFD&dbname=CJFD2010&filename=CLXW201001013&uniplatform=OVERSEA&v=2cQwTbvtJLxPfcQhjrLggvO_GuN99ClSjGNZrk4cBq04UInaW4c0d7CuMuZrnhTZ) |
| *17* | [Adolescents’ Emotional Resilience Questionnaire (AERQ)](https://doi.org/10.1007/s12144-020-00745-w) |
| *18* | [特殊儿童家长心理弹性问卷 [Psychological Resilience Questionnaire for Parents of “Special Children”]](https://chn.oversea.cnki.net/KCMS/detail/detail.aspx?dbcode=CJFD&dbname=CJFDLAST2019&filename=YXSH201901031&uniplatform=OVERSEA&v=NB8lsV9BgOLb7ezXqPI3kIYbEfMzi10wyTiJqHUQuzJXlk2QEZ07CLU50vXfUB0-) |
| *19* | [Unnamed, 9-item resiliency scale for Chinese healthcare workers in a specific study by Siu et al. (2009) (doi:10.1016/j.jrp.2009.06.008)](https://doi.org/10.1016/j.jrp.2009.06.008) |
| *20* | [The Chinese Mental Resilience Scale (CMRS)](https://doi.org/10.1177/1359105314554474) |
| *21* | [7 items from the Chinese Longitudinal Healthy Longevity Survey (CLHLS) selected for measuring resilience](https://doi.org/10.1093/geronb/gbw071) |
| *22* | [Brief Resilient Coping Scale (BRCS)](https://doi.org/10.1007/s11896-018-9252-6) |
| *23* | [Employee Resilience Measure](https://doi.org/10.1111/1744-7941.12026) |
| *24* | [Resilience and Youth Development Module (RYDM) scale, as part of the California Health Kids Survey (CHKS)](http://dx.doi.org/10.1016/j.puhe.2015.07.018) |
| *25* | [医护人员抗逆力预测试问卷/Questionnaire about Resilience of Medical Staff](https://chn.oversea.cnki.net/KCMS/detail/detail.aspx?dbcode=CJFD&dbname=CJFDLAST2016&filename=TJLT201602022&uniplatform=OVERSEA&v=KdPTEx7RNqr0uBorKbJ76e4In8NaFHV1MQsm0xxcxtDUlcHSJsS5cmrMfxScnmc6) |
| *26* | [少数民族青少年韧性素质量表 [Resilience Scale for Ethnic Minority Adolescents]](https://chn.oversea.cnki.net/KCMS/detail/detail.aspx?dbcode=CJFD&dbname=CJFD2013&filename=YJSY201305019&uniplatform=OVERSEA&v=zgqTGuUgaUdpy6w7RaUxAEcP1fARkSKB0aod81boJtA2yoPzyGQlN7vaKGb-qzls) |
| *27* | [Suicide Resiliency Inventory (SRI)](https://doi.org/10.1080/13811118.2019.1690607) |
| *28* | [Stress Resilience Quotient Scale (SRQS)](https://chn.oversea.cnki.net/KCMS/detail/detail.aspx?dbcode=CJFD&dbname=CJFDLAST2019&filename=ZLXZ201904064&uniplatform=OVERSEA&v=j9Wok-VzFZ1RLo_QwpTxASwoOjHQ6Bx9vrcCWC5WoMK_nhwEFisCEfa1Sm1gE_Ys) |
| *29* | [Resiliency Scale for Young Adults (RSYA)](https://doi.org/10.1002/pchj.256) |
| *30* | [大学生复原力量表/Resiliency Scale of University Students](https://chn.oversea.cnki.net/KCMS/detail/detail.aspx?dbcode=CJFD&dbname=CJFD2009&filename=ZLCY200903031&uniplatform=OVERSEA&v=4_yIU436NWaT3oJQfE02Zl6reDjcDl-N2oyY25Q30dq77lE3UDPdDLB_RJrKob1f) |
| *31* | [Acceptance and Action Questionnaire (AAQ)](https://chn.oversea.cnki.net/KCMS/detail/detail.aspx?dbcode=CJFD&dbname=CJFDLAST2020&filename=SXHZ202007040&uniplatform=OVERSEA&v=67DlRBz4-bpd2Gco7spX2zRIXCW82-kZuDBr1m5uq0XfRYI2n2hGBwUd5eW7QZJw) |
| *32* | [Career Motivation Measure, resilience domain](https://chn.oversea.cnki.net/KCMS/detail/detail.aspx?dbcode=CJFD&dbname=CJFDLAST2019&filename=JCJJ201904010&uniplatform=OVERSEA&v=2uosbIfD5mPyHq0Gfuck1LtVnceZPMo8Wtmt10svz9z2WDmHiAP-Xjol0GVo9W-i) |
| *33* | [中学教师复原力量表 [Resilience Scale for Highschool Teachers]](https://chn.oversea.cnki.net/KCMS/detail/detail.aspx?dbcode=CJFD&dbname=CJFDLAST2019&filename=GDSZ201806007&uniplatform=OVERSEA&v=uqhXEO-n1bEzongVc6gfEgQXOTM5yHVceExs2iEJfW49BDO2k-3GBdadqzksuK0L) |
| *34* | [Academic Resilience Scale](https://chn.oversea.cnki.net/KCMS/detail/detail.aspx?dbcode=CJFD&dbname=CJFDLAST2015&filename=JXSH201509041&uniplatform=OVERSEA&v=Bxx-gF1ndBkES-rSegcJa86c2-jdx9Z-RqW6rO6EIfadXG_BX5D3h8NqScOnpI93) |
| *35* | [Body-Mind-Spirit Well-Being Inventory 3 resilience items](https://doi.org/10.1080/13607863.2019.1584789) |
| *36* | [ClassMaps Survey (CMS) (student resilience in school settings)](https://doi.org/10.1177/0143034316664391) |
| *37* | [15-item Dispositional Resilience Scale (DRS-15)](https://doi.org/10.1080/13607863.2018.1550629) |
| *38* | [Diabetes Strengths and Resilience Measure-Teen (DSTAR-Teen)](https://doi.org/10.1111/jocn.15033) |
| *39* | [Employee Resilience Scale (EMPRES)](https://doi.org/10.3389/fpsyg.2019.00673) |
| *40* | [Psychological Performance Inventory (PPI)](https://chn.oversea.cnki.net/KCMS/detail/detail.aspx?dbcode=CJFD&dbname=CJFDLAST2015&filename=YDYX201507012&uniplatform=OVERSEA&v=KGg0ep712BvkSjhGDhCSlCnmMH2apmuVYsevIso_7mH9WZ6Cn74kX_JNwlfocvLm) |
| *41* | [Resilience Appraisals Scale (RAS)](http://dx.doi.org/10.1016/j.ajp.2015.01.011) |
| *42* | [Resilience Questionnaire](https://doi.org/10.1016/j.neuropsychologia.2018.12.002) |
| *43* | [Resilience Style Questionnaire](https://doi.org/10.1177/1073191116683798) |
| *44* | [Resilience Scale for Nurses (RSN)](https://chn.oversea.cnki.net/KCMS/detail/detail.aspx?dbcode=CJFD&dbname=CJFDLAST2020&filename=JFHL202008013&uniplatform=OVERSEA&v=cwdlFsb3RTj63h7ylqWGt97ovFuq4rxG2HeM5CgIZBk-nqS-JeIzS0qnsQ91PDpG) |
| *45* | [运动员心理韧性问卷/The Questionnaire of Athletes’ Resilience](https://chn.oversea.cnki.net/KCMS/detail/detail.aspx?dbcode=CJFD&dbname=CJFDLAST2015&filename=TYYK201505015&uniplatform=OVERSEA&v=LO47mkVmL0f3YSGGJmlleBDfuDWVOZiEuDHX-zeoBpfDeDlZHhbETKnoIIZ_Tum3) |
| *46* | [中学生心理健康自评量表 [Self-Report Mental Health Scale for Highschool Students] resilience items](https://chn.oversea.cnki.net/KCMS/detail/detail.aspx?dbcode=CJFD&dbname=CJFD2008&filename=PJYC200803012&uniplatform=OVERSEA&v=mU20iKCjAIPUmhZRRVMNa-G654laddyB29VY61WHwyXZVZNNKcTzttYx_wwf7uN6) |
| *47* | [优秀运动员心理韧性量表/Psychological Resilience Scale for Elite Athletes](https://chn.oversea.cnki.net/KCMS/detail/detail.aspx?dbcode=CJFD&dbname=CJFD2012&filename=TJTY201204021&uniplatform=OVERSEA&v=oMevvBYmlfYzqBo9cJpu3iGn32GWYBDx_3fKeV9UTDoUOCOmro0tbHJ_lyVJko91) |
| *48* | [青少年抗逆力测量中文版 [Chinese Resilience Measure for Adolescents]](https://chn.oversea.cnki.net/KCMS/detail/detail.aspx?dbcode=CJFD&dbname=CJFD2013&filename=ZGQL201306014&uniplatform=OVERSEA&v=5UuReEfOxmlz1fBY93s2d59BqmbmfKN5WIe3A1RKIM6fqkWQ6vbdgHy_gZMd67f4) |
| *49* | [韧性量表/Resilience Scale (not RS)](https://chn.oversea.cnki.net/KCMS/detail/detail.aspx?dbcode=CJFD&dbname=CJFD2011&filename=ZXWS201105015&uniplatform=OVERSEA&v=UZ_CtOqBkq4rJbJ-HzMRnROL9cpcih1hEWqVL0UbP1qE1poXXF-2yDoil4-EyrD7) |
| *50* | [中国空巢老人的心理弹性量表 [Psychological Resilience Scale for Chinese Empty Nesters]](https://chn.oversea.cnki.net/KCMS/detail/detail.aspx?dbcode=CJFD&dbname=CJFDLAST2015&filename=ZLXZ201519110&uniplatform=OVERSEA&v=hdk8Gjl8utoU2k5ZJI80GVbka81jd54RA1U1-4hexRBuBtPDJtJ0bkxGi1E-1MVD) |
| *51* | [Sukemune-Hiew Resilience Test (in Japanese)/S-H式心理弹性检查量表](https://chn.oversea.cnki.net/KCMS/detail/detail.aspx?dbcode=CJFD&dbname=CJFDLAST2019&filename=YJSY201904015&uniplatform=OVERSEA&v=lwR96WhiXUZ6_x_zyDdeYr16qRkG-I88D0FpOvbJ9NWWFiIUNLhCcrTAiKO9hwKC) |
| *52* | [随迁子女复原力量表/The Resiliency Questionnaire of Migrant Worker's Children](https://chn.oversea.cnki.net/KCMS/detail/detail.aspx?dbcode=CJFD&dbname=CJFDLAST2015&filename=ZDTJ201507011&uniplatform=OVERSEA&v=PyD1nD6JzcK_AunPYmoC5TBwTToH-56flWklkKcSPj14ZDTugswdVyB7PoeU8HnV) |
| *53* | [幼儿教师心理弹性问卷 [Psychological Resilience Questionnaire for Preschool Teachers]](https://chn.oversea.cnki.net/KCMS/detail/detail.aspx?dbcode=CJFD&dbname=CJFDLAST2018&filename=CLXW201804012&uniplatform=OVERSEA&v=ZDdiIahewQ2rHmviqjkwy1rLYo67dl017O29-05rOs2JK0jXBOqkveS9O94-GaiF) |
| *54* | [老年人心理弹性量表 [Resilience Scale for Elders] (based on RSA)](https://chn.oversea.cnki.net/KCMS/detail/detail.aspx?dbcode=CJFD&dbname=CJFDLAST2015&filename=QKYX201507027&uniplatform=OVERSEA&v=MprlmDKX9_PDZyBZ6QCKAeNZKu0aLhqb58UNwaOqX4lYzmgf2nfAgaB4RvcRn3TQ) |
| *55* | [Psychological Response to Post Disaster Situation resilience items](https://doi.org/10.1080/21683603.2016.1254131) |
| *56* | [Unnamed, self-developed resilience scale for financial managers](https://chn.oversea.cnki.net/KCMS/detail/detail.aspx?dbcode=CJFD&dbname=CJFDLAST2015&filename=XDGL201505006&uniplatform=OVERSEA&v=R-jx0VH3FCBxQ5Tz90dIX7x7Q01LkxiafhWuBBFfFYhNLDSeTkp3g3jh73iPCv_6) |
| *57* | [Resilience Attribute Scale](https://doi.org/10.1002/casp.2340) |
| *58* | [Adolescent Psychological Resilience Questionnaire](https://doi.org/10.2224/sbp.6382) |
| *59* | [大学生心理弹性问卷 [Resilience Questionnaire for College Students]](https://chn.oversea.cnki.net/KCMS/detail/detail.aspx?dbcode=CJFD&dbname=CJFDLAST2019&filename=ZWSG201812022&uniplatform=OVERSEA&v=S3N_CdnsUoDSVeAi1_azDkoO3R5czvjMNjjoHdUXkovBfylEmJKlxUhQchIB5aFK) |
| *60* | [Resilience Inventory](https://chn.oversea.cnki.net/KCMS/detail/detail.aspx?dbcode=CJFD&dbname=CJFDLAST2018&filename=BJSF201804003&uniplatform=OVERSEA&v=GtDjU8PnUjXWiKUb7qdhIPzH9GuKi5tOVnLn50yIccLjt8RHiYoRMcet5B5G0B6X) |
| *61* | [Adolescent Resilience Scale-Chinese Version](https://doi.org/10.1177/1367493514540815) |
| *62* | [心理弹性量表/Psychological Resilience Scale (PRS-25)](https://chn.oversea.cnki.net/KCMS/detail/detail.aspx?dbcode=CJFD&dbname=CJFDLAST2017&filename=ZLXZ201710091&uniplatform=OVERSEA&v=4ooJAQQW7VEeOg9RHvqG1YB_fazGDyyIPnM1vjKxQhPZCVI5_wjFHceoE50XQ7j5) |
| *63* | [中学生复原力量表 [Resilience Scale for Middle School Students]](https://chn.oversea.cnki.net/KCMS/detail/detail.aspx?dbcode=CJFD&dbname=CJFDLAST2015&filename=JXGL201506025&uniplatform=OVERSEA&v=dfViJ5-gFPzDCfRNYZYG45m55w5I9M3xG_a2uGnG9mbJsaEHgy24yXHl3QCDNvQp) |
| *64* | [Academic Resilience Questionnaire for Secondary Vocational Students](https://chn.oversea.cnki.net/KCMS/detail/detail.aspx?dbcode=CJFD&dbname=CJFDLAST2020&filename=ZYJW201931013&uniplatform=OVERSEA&v=Ww4B-K-Byupn8AJ_MmOIWbLBcrut6N9skMKWLR8746Mj4L3lHT8X5N2Dp1bzShEH) |
| *65* | [Academic Resilience Scale (ARS-30)](https://doi.org/10.1016/j.childyouth.2020.105029) |
| *66* | [Children’s Hope Scale (CHS)](https://chn.oversea.cnki.net/KCMS/detail/detail.aspx?dbcode=CJFD&dbname=CJFDLAST2022&filename=NYSK202002010&uniplatform=OVERSEA&v=lbi04R6WHqYwGCNY5Ro0CeqaF90nF2qtRfot3Z9wKJioh8MNUo2V-IttteQKEFyf) |
| *67* | [Inventory of Adolescent Resilience](https://doi.org/10.3389/fpsyg.2020.568906) |
| *68* | [Unnamed set of items for measuring vocational schoolteachers’ resilience](https://chn.oversea.cnki.net/KCMS/detail/detail.aspx?dbcode=CJFD&dbname=CJFDLAST2018&filename=ZJLT201808014&uniplatform=OVERSEA&v=8Z_bmj3jaao3QSJ5tmWrAyua_EJWGXoS36DiEXsK6D0yd6I3hWjoRRi7qAPXkRBi) |
| *69* | [脑卒中患者康复复原力问卷 [Rehabilitation Resilience Questionnaire for Stroke Patients]](https://chn.oversea.cnki.net/KCMS/detail/detail.aspx?dbcode=CJFD&dbname=CJFDLAST2020&filename=HLXZ202015007&uniplatform=OVERSEA&v=VBqQdSxO7bV-tG8ZwT0CeJ8YK9XDPyo4nDtRs32LdZYSvQkiqx0DheN-R0h3dzR-) |
| *70* | [Unnamed set of items about health activity participation of elders as a resilience measure](https://chn.oversea.cnki.net/KCMS/detail/detail.aspx?dbcode=CJFD&dbname=CJFDLAST2020&filename=YNZZ202004011&uniplatform=OVERSEA&v=MxAl_b5SHv84GqUgn-JFZLhu2chxjbE39HlvnzVozYG0oUdZMUiZNCD6hy8FKoQT) |
| *71* | [针对危机救援人员编制的抗逆力问卷/Measurement of Resilience of Crisis Rescuer in China](https://chn.oversea.cnki.net/KCMS/detail/detail.aspx?dbcode=CJFD&dbname=CJFD2014&filename=XIAO201401009&uniplatform=OVERSEA&v=e7imx8eu4DHZHbwqUuegpfiLMbAtw7q005pWmR447JO_HGW4V5HVcsPw2n8Gck10) |
| *72* | [Career Evolution Capacity Inventory resilience subscale](https://doi.org/10.1017/prp.2015.13) |
| *73* | [老年人心理弹性量表/Elderly Resilience Scale](https://chn.oversea.cnki.net/KCMS/detail/detail.aspx?dbcode=CJFD&dbname=CJFDLAST2019&filename=ZLXZ201906066&uniplatform=OVERSEA&v=j9Wok-VzFZ0IiMxmX2MpaWUS9pACf__YSaPKOIgwXJo8Hnd0WWx3P9NiuUJN8hFn) |
| *74* | [Attitudes towards Recovery Questionnaire (ARQ) resilience as a person in recovery domain](https://doi.org/10.1016/j.psychres.2018.05.047) |
| *75* | [A multivariate approach to measuring resilience, indicated by academic performance, mental health, and prosocial behavior](https://doi.org/10.1002/jcop.22303) |
| *76* | [The Chinese version of the Resilience Scale (not RS) for Southeast Asian immigrant women who divorced in Taiwan](https://doi.org/10.1371/journal.pone.0211451) |
| *77* | [Workforce Agility Measure 4 resilience items](http://dx.doi.org/10.1016/j.ijinfomgt.2017.09.001) |
| *78* | [The Resilience Scale of University Students (RSUS)](https://doi.org/10.1016/j.nepr.2019.102624) |
| *79* | [Unnamed set of self-reported resilience-oriented strategies in flooding zones](https://doi.org/10.3390/ijerph16142559) |
| *80* | [Specific Scale Adapted from BRS and RSA](https://doi.org/10.1080/17441692.2016.1240822) |
| *81* | [Scale made of items from both CD-RISC-25 and RSCA](https://doi.org/10.1089/cyber.2017.0319) |
| *82* | [The Pain Resilience Scale (PRS)](https://doi.org/10.1111/joor.12591) |
| *83* | [Career Motivation Scale (CMS) – seven career resilience items](https://pubmed.ncbi.nlm.nih.gov/31547475/) |
| *84* | [Asian Resilience Scale (ARS)](https://doi.org/10.1186/s40359-020-00444-y) |
| *85* | [“Academic Buoyancy” 4 items](https://chn.oversea.cnki.net/KCMS/detail/detail.aspx?dbcode=CJFD&dbname=CJFDLAST2015&filename=JXSH201509041&uniplatform=OVERSEA&v=Bxx-gF1ndBkES-rSegcJa86c2-jdx9Z-RqW6rO6EIfadXG_BX5D3h5H3cgsrTZY_) |
